# A High-Sensitivity MXene/PVDF Flexible Piezoelectric Sensor for Intelligent Tunnel Lighting

**DOI:** 10.3390/ma19101976

**Published:** 2026-05-11

**Authors:** Xi Xiong, Long Jin, Shenglong Wang, Tianpei Xu, Jiabin Zhang, Longchao Huang, Yong Ao, Weili Deng, Weiqing Yang

**Affiliations:** 1 Key Laboratory of Advanced Technologies of Materials (Ministry of Education), School of Materials Science and Engineering, Southwest Jiaotong University, Chengdu 610031, China; xixiong@my.swjtu.edu.cn (X.X.); yzbydq@my.swjtu.edu.cn (S.W.); tianpeixu@my.swjtu.edu.cn (T.X.); 2433773945@my.swjtu.edu.cn (J.Z.); hlc@my.swjtu.edu.cn (L.H.); ayy@my.swjtu.edu.cn (Y.A.); weili1812@swjtu.edu.cn (W.D.); wqyang@swjtu.edu.cn (W.Y.); 2 Research Institute of Frontier Science, Southwest Jiaotong University, Chengdu 610031, China

**Keywords:** MXene, PVDF, electrohydrodynamic printing, piezoelectric sensor

## Abstract

Polyvinylidene fluoride (PVDF), one of the most promising flexible piezoelectric polymers bridging mechanical compliance and infrastructure-scale sensing, suffers from low intrinsic β-phase content that limits energy conversion efficiency. Two-dimensional MXene nanosheets offer a compelling solution, inducing β-phase crystallization through interfacial hydrogen bonding while preserving essential flexibility, yet conventional fabrication methods lack precise control over dipole alignment and suffer from percolation leakage at functional loadings. Herein, we report a process-structure synergistic strategy that combines EHD printing with an optimized serpentine structure to reconcile piezoelectric sensitivity with mechanical durability. By precisely tuning the MXene loading to 0.75 wt% (near but below the percolation threshold), the composite achieves a β-phase content of 71.91% and a piezoelectric sensitivity of 18.09 mV/kPa, while the serpentine design delivers a tensile strength of 21.97 MPa and 17.46% elongation at break. As a proof-of-concept, the sensor is deployed in a vehicle-responsive tunnel lighting system, withstanding cyclic heavy loads and achieving a 95.04% energy-saving rate compared to continuous operation. This work advances high-performance flexible piezoelectric composites for intelligent infrastructure applications.

## 1. Introduction

Piezoelectric materials that convert mechanical energy into electrical signals have become a research hotspot in self-powered sensing and energy harvesting. Polyvinylidene fluoride (PVDF) is one of the most promising piezoelectric polymers due to its ultrahigh flexibility, excellent chemical stability, and processability [1,2,3,4]. The piezoelectricity of PVDF originates from the all-trans (TTTT) conformation β-phase with aligned dipoles perpendicular to the c-axis, which exhibits the maximum spontaneous polarization intensity [5,6,7,8]. However, pure PVDF suffers from low intrinsic β-phase content and random dipole arrangement, leading to limited piezoelectric performance, which restricts its practical application [9,10,11]. While incorporating ceramic fillers such as barium titanate or zinc oxide can enhance polarization, these rigid additives inevitably compromise mechanical flexibility and fatigue resistance due to modulus mismatch and poor interfacial compatibility [12,13,14,15]. Although other 2D nanofillers such as graphene have also been explored to improve the piezoelectric performance of PVDF composites, their limited polar functional groups fail to induce sufficient β-phase crystallization and strong interfacial interactions with the polymer matrix [16,17]. Alternatively, two-dimensional (2D) Ti_3_C_2_T_x_ MXene nanosheets offer a distinct advantage by combining high electrical conductivity, large specific surface area, and inherent flexibility. Their abundant surface functional groups (-OH) can form hydrogen bonds with PVDF molecular chains, thereby inducing β-phase crystallization and promoting dipole alignment without significantly sacrificing mechanical compliance [18,19,20]. Nevertheless, traditional fabrication methods (e.g., solution casting, electrospinning) lack precise control over interfacial polarization orientation and spatial filler distribution, often resulting in random dipole arrangements, agglomeration-induced percolation leakage at higher loadings, and inability to achieve programmable patterning for complex device geometries [21,22,23,24].

EHD printing theoretically resolves these limitations by generating strong electric fields during deposition that simultaneously enable pattern precision and promote dipole orientation in situ [25,26]. While EHD printing-induced electric fields enhance β-phase crystallinity for superior piezoelectric output, the resulting high crystallinity typically increases stiffness and brittleness, rendering the composite vulnerable to structural failure under severe mechanical deformation [27,28,29]. Conversely, adopting flexible serpentine geometries to improve stretchability risks disrupting the uniform electric field distribution required for consistent polarization during EHD processing, or inducing interfacial delamination between MXene and PVDF layers under cyclic strain, thereby degrading piezoelectric performance [30,31,32]. Furthermore, the conductive nature of MXene establishes a percolation paradox: loadings sufficient for effective heterogeneous nucleation approach the threshold where compressive strain promotes charge leakage pathways, creating an unresolved trade-off between piezoelectric sensitivity and electrical insulation [33,34]. These conflicting constraints fundamentally restrict the operational envelope of such composites, preventing reliable deployment in cyclic heavy-load environments demanding simultaneous high sensitivity, mechanical durability, and long-term fatigue resistance.

In this work, we report a process-structure synergistic strategy that overcomes these material limitations. By precisely optimizing the MXene loading to 0.75 wt%, near but below the percolation threshold, we leverage EHD printing to achieve concurrent enhancement of β-phase crystallinity (71.91%) and controlled dipole orientation, yielding a high piezoelectric sensitivity of 18.09 mV/kPa. Critically, we introduce an optimized serpentine-structure design that decouples the mechanical-electrical trade-off: the geometry disperses mechanical stress to prevent brittle failure while maintaining the integrity of the polarized microstructure, delivering both high tensile strength (21.97 MPa) and superior flexibility (elongation at break 17.46%). As a validation of practical viability under severe cyclic loading conditions, the sensor system is deployed in a vehicle-responsive tunnel lighting application, targeting the substantial energy waste of conventional continuous illumination in low-traffic environments. The system withstands repeated heavy mechanical stress and achieves a remarkable energy-saving rate of 95.04%. This work presents a synergistic approach that advances the design of high-performance piezoelectric composites, providing new insights into balancing piezoelectric sensitivity with mechanical durability for infrastructure-integrated sensing applications.

## 2. Experimental Section

### 2.1. Preparation of MxeneDMF

First, 30 mL of 12 M hydrochloric acid (HCl, Sigma-Aldrich, St. Louis, MO, USA) was diluted with 10 mL of ultrapure water to prepare 40 mL of 9 M HCl solution. Subsequently, 2 g of lithium fluoride (LiF, Sigma-Aldrich, St. Louis, MO, USA) was dissolved in the as-prepared HCl solution under magnetic stirring for 10 min to form a homogeneous etching mixture. Then, 2 g of MAX phase powder (400 mesh, 11 Technology Co., Ltd., Shanghai, China) was slowly added to the LiF/HCl solution, and the reaction mixture was stirred in a water bath at 40 °C for 24 h at 1000 rpm to fully etch the Al layer. Then, the reaction product was centrifuged (3500 rpm, 5 min) and washed repeatedly with deionized water until the pH of the supernatant exceeded 6. Afterward, the wet MXene precipitate was redispersed in N, N-dimethylformamide (DMF), followed by centrifugation at 5000 rpm for 3 min. This centrifugation-redispersion cycle was repeated seven times to exfoliate MXene into few-layer or single-layer nanosheets, significantly improving its dispersibility in organic solvents. Finally, a stable MXene/DMF dispersion was obtained, and its concentration was calibrated as 20 mg/mL by vacuum-drying a small aliquot of the dispersion.

### 2.2. Preparation of MxenePVDF Composites via EHD Printing

MXene/PVDF inks with different MXene mass fractions (0.25, 0.50, 0.75, 1.00, and 1.25 wt%, relative to PVDF mass) were prepared. First, 1.2 g of PVDF (Archma, Colombes, France) was dissolved in a mixed solvent of MXene/DMF/Acetone. The mixture was stirred at 55 °C for 4 h to obtain a homogeneous composite ink suitable for EHD printing. The composite films were fabricated using an EHD printing system. The prepared ink was loaded into a syringe equipped with a stainless steel needle (inner diameter = 0.34 mm). The optimized printing parameters were set as follows: printing speed = 0.8 mm/s, applied high voltage = 3 kV, substrate = ITO conductive glass, printing distance (needle tip to substrate) = 1 cm, and ambient temperature = 32.5 °C. After printing, the as-deposited composite films were dried in an oven at 55 °C for 2 h to remove residual organic solvents.

### 2.3. Fabrication of Piezoelectric Sensors

First, silver (Ag) electrodes were sputtered on both sides of the composite films via magnetron sputtering (Ag target purchased from ZhongNuo Advanced Material (Beijing) Technology Co., Ltd., Beijing, China). Then, conductive carbon strips were then pasted on the Ag electrodes. Afterward, the piezoelectric sensor was tightly encapsulated with silicone rubber to protect the electrodes from mechanical damage.

### 2.4. Characterization of Composites and Sensors

The SEM (JSM7800F, JEOL Ltd., Tokyo, Japan) was used to characterize the morphology of MXene/PVDF composites. The XPS (ESCALAB XI +, Thermo Fisher Scientific, Waltham, MA, USA) was applied to study the surface elemental composition and chemical bonding states of MXene. The XRD (Empyrean, PANalytical, Almelo, The Netherlands) and DSC (TGA/DSC 3+, Mettler Toledo, Columbus, OH, USA) and FTIR (Nicolet 5700 Spectrometer, Thermo Fisher Scientific, Waltham, MA, USA) were applied to study the phase structure and crystal content of the MXene/PVDF.

The periodic normal force with a frequency of 2 Hz and an acceleration of 0.5 m/s^2^ was applied to the sensors using a linear motor (LinMot H01-23 × 86/160, LinMot, Spretenbach, Switzerland) and a force gauge (IMADA model ZPS-DPU-50 N, IMADA, Aichi, Japan). The piezoelectric outputs (open-circuit voltage *V*_oc_, short-circuit current *I*_sc_) were measured by a programmable electrometer (Keithley 6517, Keithley Instruments, Cleveland, OH, USA) at a sampling rate of 1000.

## 3. Results and Discussion

### 3.1. Fabrication and Design of Piezoelectric Sensors

The serpentine-structure MXene/PVDF flexible piezoelectric sensors are fabricated via EHD printing technology. The preparation process mainly consists of two core steps: mixing MXene with PVDF to obtain a homogeneous composite ink, and performing EHD printing under a high-voltage electric field to acquire patterned serpentine-structure composite films. The etching of MXene and the detailed preparation process of the sensors are discussed in the Section 2.

**Figure 1:**
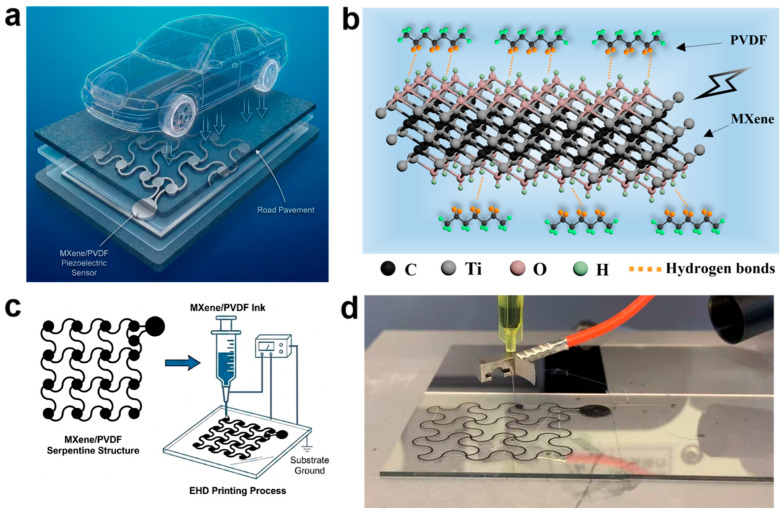
Concept and design of MXene/PVDF flexible piezoelectric sensors for intelligent perception of vehicles. (**a**) Schematic illustration of a pavement-integrated MXene/PVDF piezoelectric sensor. (**b**) Molecular model diagram of hydrogen bond formation via the interaction between MXene and β-PVDF under electric polarization. (**c**) Fabrication process of the serpentine-structure MXene/PVDF composite films via the EHD printing. (**d**) Photograph of the EHD printing fabrication process.

Figure 1a illustrates the overall concept of the pavement-integrated MXene/PVDF piezoelectric sensor and its application scenario in intelligent vehicle perception for tunnel lighting. The mechanical pressure exerted by passing vehicles is converted into electrical signals to trigger on-demand control of the lighting. Figure 1b presents the molecular model of the interaction between MXene and β-PVDF under electric polarization. The functional groups (-OH) on the surface of MXene form strong hydrogen bonds with the -CH_2_-/-CF_2_- dipoles of PVDF molecular chains, thereby promoting the directional alignment of dipoles and the crystallization of the piezoelectrically active β-phase [35,36,37,38,39].

During the EHD printing process (Figure 1c,d and Figure S2), the MXene/PVDF composite ink is extruded from the nozzle under a high-voltage electric field, forming a stable Taylor cone (Figure S2a,b), and finally deposited on the ITO conductive glass substrate to form well-defined serpentine patterns (Figure 1d and Figure S2c). This deliberately designed serpentine structure can confine the stress distribution under repeated vehicle loads, endowing the sensor with excellent mechanical robustness and flexibility, which is critical for reliable integration into road pavement.

Then, to realize the practical application of the sensor in tunnel lighting, an intelligent sensing system based on MXene/PVDF piezoelectric sensors is designed and fabricated (Figure S1). As shown in Figure S1a, when a vehicle travels over the sensor-embedded road pavement, the under-pavement sensor panel converts the mechanical pressure into piezoelectric pulse signals, triggering the activation of tunnel lighting in the area ahead of the vehicle. The electrical control block diagram of the system is depicted in Figure S1b, after the piezoelectric pulse signals are processed by the IC board, they are transmitted to the Programmable Logic Controller (PLC) via RS485 communication, which then controls the AC contactor to realize the intelligent switching of the lighting group, ensuring the lighting remains on for a preset duration to allow the vehicle to completely exit the illuminated area.

### 3.2. Characterizations of Mxene/PVDF Composite Films

**Figure 2:**
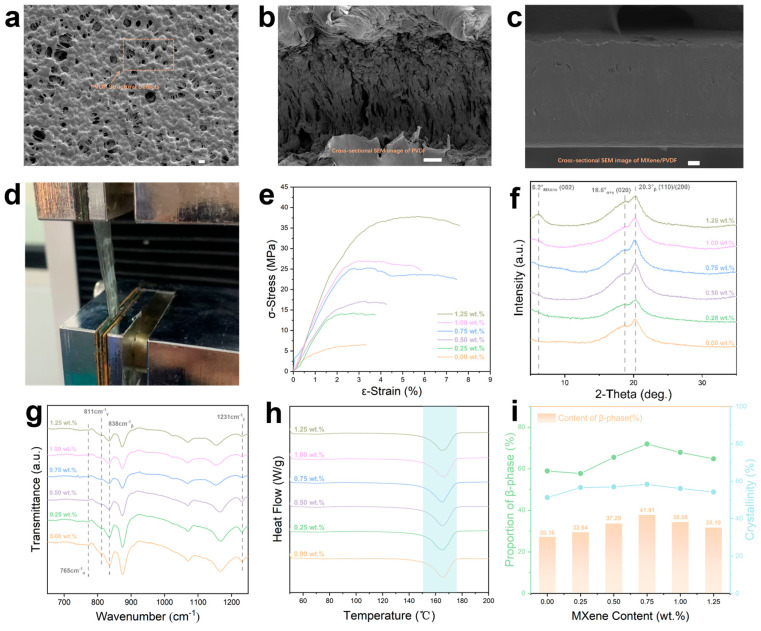
Characterizations of the MXene/PVDF composite films. (**a**) Surface SEM image of pure PVDF (scale bar is 10 μm). (**b**) Cross-sectional SEM image of pure PVDF (scale bar is 10 μm). (**c**) Cross-sectional SEM image of 0.75 wt% MXene/PVDF (scale bar is 10 μm). (**d**) Photograph of the tensile test process for stress–strain measurement. (**e**) Stress–strain curves of MXene/PVDF with different MXene contents. (**f**) XRD patterns of MXene/PVDF with different MXene contents. (**g**) FTIR spectra of MXene/PVDF with different MXene contents. (**h**) DSC thermographs of MXene/PVDF with different MXene contents. (**i**) Crystallinity, proportion and content of the β-phase of MXene/PVDF with different MXene contents, quantified from the DSC thermographs and the FTIR spectra.

The surface and cross-sectional morphologies of pure PVDF and MXene/PVDF composite films were investigated by SEM (Figure 2a–c and Figure S3). As shown in Figure 2a and Figure S3a, the surface of the pure PVDF film exhibits a porous structure with uniformly distributed micro-pores, while the corresponding cross-sectional image (Figure 2b and Figure S3b) reveals a distinct layered morphology with clear phase separation, an inherent feature of solution-cast PVDF that leads to relatively poor mechanical robustness. For the MXene/PVDF composite films, the microstructure evolves significantly with increasing MXene content. The composite film with 0.25 wt% MXene (Figure S3c) shows a relatively smooth surface with reduced porosity compared to pure PVDF, while the 0.50 wt% MXene film (Figure S3d) displays a slightly rougher surface with more pronounced microstructures. Notably, the 0.75 wt% MXene/PVDF composite film (Figure 2c and Figure S3e) exhibits a dense and uniform surface (left and middle panels) and a compact cross-sectional structure (right panel) without obvious agglomeration, confirming the excellent dispersion of MXene nanosheets within the PVDF matrix at this loading. Further increasing the MXene content to 1.00 wt% and 1.25 wt% (Figure S3f) results in a more pronounced layered cross-sectional structure with increased filler dispersion, yet subtle agglomeration can be observed, indicating that excessive MXene loading may introduce structural defects. These morphological changes confirm that the MXene content critically regulates the composite microstructure, which is foundational for optimizing piezoelectric and mechanical performance.

The chemical composition and surface elemental states of as-etched MXene are characterized by XPS (Figure S4). The high-resolution XPS spectra of C 1s, O 1s, and Ti 2p were collected to confirm the successful preparation of MXene. The C 1s spectrum (Figure S4a) shows a dominant peak at ~284.8 eV, corresponding to C–C bonds in the MXene lattice. The O 1s spectrum (Figure S4b) exhibits a strong peak at ~532.0 eV, assigned to surface oxygen-containing functional groups (e.g., –OH) introduced during the etching of the MAX phase. The Ti 2p spectrum (Figure S4c) displays two characteristic peaks at ~455.5 eV (Ti-C bonds) and ~461.5 eV (Ti-O bonds), confirming the successful preparation of MXene nanosheets with abundant surface functional groups. These -OH groups are critical for forming strong hydrogen bonds with PVDF’s -CH_2_-/-CF_2_- dipoles, which promotes dipole alignment and β-phase crystallization in the composite.

The crystalline structures of pure MXene, pure PVDF, and MXene/PVDF composites were analyzed by XRD (Figure 2f and Figure S5). Pure MXene (Figure S5) shows a distinct (002) diffraction peak at 2θ = 6.2°, a signature of successfully exfoliated nanosheets (confirming Al layer removal from the MAX phase). Pure PVDF exhibits a broad peak at 2θ = 20.3°, corresponding to overlapping (110)/(200) planes of its α and β phases [40,41]. In MXene/PVDF composites (Figure 2f), the MXene (002) peak is not prominent—attributed to low MXene loading and good dispersion—while the PVDF peak shows a slight shift and intensity change, indicating strong interfacial interactions between MXene and PVDF that modify PVDF’s crystalline structure.

FTIR spectroscopy (Figure 2g) was used to quantify phase composition. The peak at 765 cm^−1^ corresponds to the non-piezoelectric α-phase, the peak at 838 cm^−1^ corresponds to the piezoelectric β-phase, and the peaks at 811 cm^−1^ and 1231 cm^−1^ correspond to the semi-piezoelectric γ-phase [42,43]. with increasing MXene content, the peak intensities of the β-phase and γ-phase gradually increase (while the peak intensity of the α-phase decreases), reaching the maximum at 0.75 wt%.

DSC (Figure 2h) was used to calculate crystallinity (*χ_c_*) via the equation:

$$\chi_{c}=\frac{\Delta H_{m}}{\Delta H_{0}}\times 100\%$$

where ΔH_m_ is the sample melting enthalpy and ΔH_0_ = 104.7 J/g is the melting enthalpy of 100% crystalline PVDF. Quantitative results for crystallinity and β-phase proportion (derived from FTIR via the Lambert–Beer equation) are shown in Figure 2i [44]. Both values first increase and then decrease with MXene content, peaking at 0.75 wt% (*χ_c_* = 58.28%, β-phase proportion = 71.91%). This trend arises because appropriate MXene loading acts as a heterogeneous nucleant to promote crystallization and β-phase formation, while excessive loading causes agglomeration that disrupts PVDF’s crystalline order.

The mechanical performance of MXene/PVDF composites was evaluated via tensile tests (Figure 2d,e). Figure 2d shows a photograph of the tensile test setup, while Figure 2e presents stress–strain curves for different MXene loadings. The tensile strength and elongation at break first increase and then decrease with MXene content, with the 0.75 wt% composite exhibiting optimal mechanical properties (σ-Stress = 25.27 MPa, ε-Strain = 7.46%)—significantly higher than pure PVDF. This enhancement stems from uniform MXene dispersion, which effectively transfers stress and reinforces the PVDF matrix. Further increasing MXene content to 1.00 wt% and 1.25 wt% reduces mechanical performance, as agglomeration introduces structural defects and weakens interfacial bonding. Combined with the serpentine-structure design, the robust mechanical properties of the 0.75 wt% composite ensure reliable sensor integration into road pavement and long-term durability under repeated vehicle loads.

### 3.3. Piezoelectric Enhancement Mechanism and Piezoelectric Properties of Mxene/PVDF Piezoelectric Sensors

**Figure 3:**
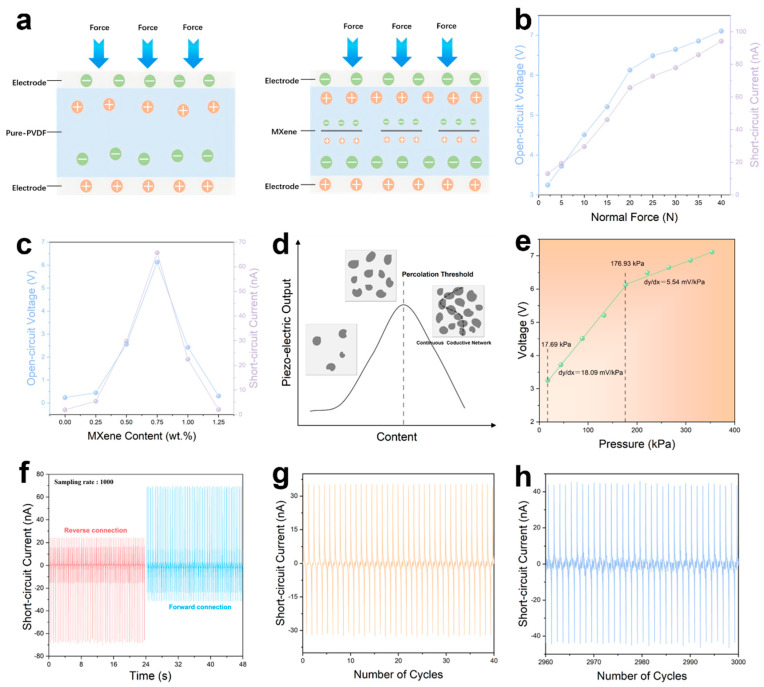
Piezoelectric enhancement mechanism and electrical output performance of MXene/PVDF piezoelectric electronics. (**a**) Schematic illustration of the MXene/PVDF interface promoting PVDF polarization and enhancing piezoelectricity. (**b**) Open-circuit Voltages and short-circuit currents of the 0.75 wt% MXene/PVDF piezoelectric electronic. (**c**) Open-circuit Voltages and short-circuit currents of the piezoelectric electronics with different MXene contents. (**d**) Schematic illustration of the piezoelectric output variation caused by the percolation effect. (**e**) Pressure sensitivities of the 0.75 wt% MXene/PVDF piezoelectric electronic. (**f**) Short-circuit currents of the 0.75 wt% MXene/PVDF piezoelectric electronic under forward and reverse connection. (**g**) The first 40 cycles short-circuit current of the 0.75 wt% MXene/PVDF piezoelectric electronic (total 3000 cycles). (**h**) The last 40 cycles Short-circuit current of the 0.75 wt% MXene/PVDF piezoelectric electronic (total 3000 cycles).

Figure 3a illustrates the piezoelectric enhancement mechanism at the MXene/PVDF interface. Under the high-voltage electric field during EHD printing, the hydrogen bonds formed between MXene and PVDF promote the polarization of PVDF and the formation of piezoelectrically active β-phase. Under the same mechanical stress, the piezoelectric performance of the MXene/PVDF composite is far superior to that of pure PVDF.

To evaluate the piezoelectric performance of devices with different MXene contents, the piezoelectric output of the devices was measured at a frequency of 2 Hz, an acceleration of 0.5 m/s^2^, and a sampling rate of 1000. As shown in Figure 3c and Figure S6, both *V*_oc_ and *I*_sc_ exhibit a trend of first increasing and then decreasing with the increase in MXene content. Among all the tested devices, the sample with 0.75 wt% MXene loading shows the best electrical performance. As presented in Figure 3b and Figure S7, the piezoelectric output of the 0.75 wt% sample under a normal force of 15 N is as follows: *V*_oc_ reaches 5.21 V and *I*_sc_ reaches 46.15 nA, while those of pure PVDF are only 0.64 V and 5.06 nA, respectively.

When the MXene content exceeds 0.75 wt%, the excess MXene nanosheets contact each other in the PVDF matrix and form continuous conductive pathways, causing internal current leakage, which destroys the effective accumulation and output of piezoelectric charges, and ultimately leads to a significant reduction in piezoelectric performance (percolation effect, Figure 3d). Under a pressure of 17.69–353.86 kPa and a frequency of 2 Hz, the output voltage of the 0.75 wt% sample increases monotonically with pressure. The pressure sensitivity is defined as the slope of the voltage-pressure curve (Figure 3e): the sensitivity in the low-pressure region (pressure < 176.93 kPa) is 18.09 mV/kPa, and that in the high-pressure region (176.93–353.86 kPa) is 5.54 mV/kPa, both showing good linearity. The short-circuit current output of the 0.75 wt% sample under forward and reverse connection was tested at a pressure of 176.93 kPa and a frequency of 2 Hz (Figure 3f). The polarity of the current reverses synchronously with the connection direction, confirming that the electrical output originates from the piezoelectric effect and the charge collection of the device is reliable. Under the same conditions, the electrical output shows no attenuation trend after 3000 cycles of the cycling stability test, proving the excellent electrical stability of the device (Figure 3g,h).

### 3.4. Serpentine-Structure MxenePVDF Flexible Sensors for Intelligent Tunnel Lighting Application

**Figure 4:**
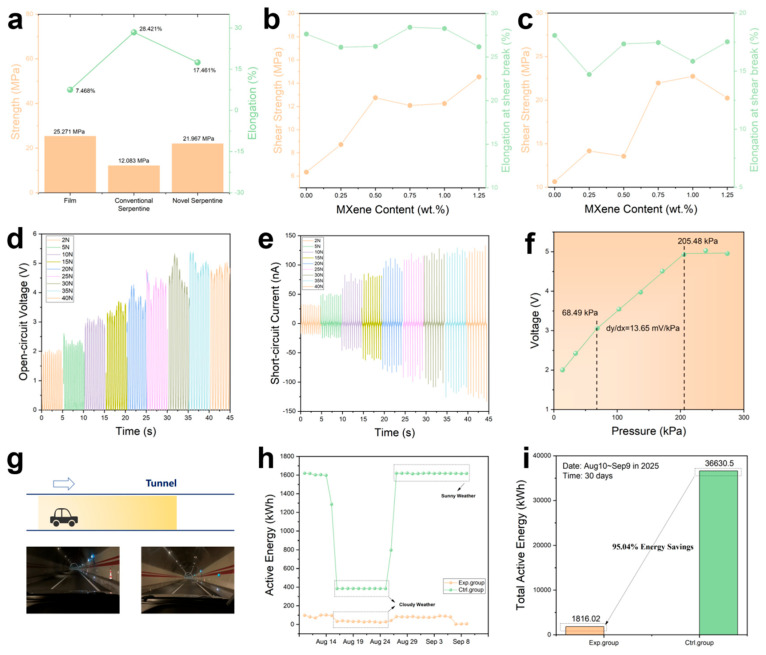
Piezoelectric output performance of serpentine-structure MXene/PVDF flexible sensors and intelligent tunnel lighting application. (**a**) Physical properties (strength and elongation) of film, conventional and novel serpentine structure (used in this work) of the 0.75 wt% MXene/PVDF composites. (**b**) Shear strength and elongation at break of the conventional serpentine structure of MXene/PVDF composites with different MXene contents. (**c**) Shear strength and elongation at break of the novel serpentine structure of MXene/PVDF composites with different MXene contents. (**d**) Open-circuit voltages of the 0.75 wt% MXene/PVDF flexible sensors. (**e**) Short-circuit currents of the 0.75 wt% MXene/PVDF flexible sensors. (**f**) Pressure sensitivities of the 0.75 wt% MXene/PVDF flexible sensors. (**g**) Schematic illustration of intelligent sensing for tunnel lighting. (**h**) Active energy consumption of the control group and experimental group (intelligent sensing). (**i**) Total energy consumption of the control group and experimental group over 30 days.

To meet the practical requirements for integration into tunnel pavements and withstand repeated vehicle loading, the optimized 0.75 wt% MXene/PVDF composite is fabricated into a serpentine structure. The mechanical properties (tensile strength and elongation at break) of the pure film, conventional serpentine structure, and the novel serpentine structure used in this work were systematically compared (Figure 4a). The film exhibits a high tensile strength of 25.27 MPa but a low elongation at break of 7.47%, indicating poor flexibility that limits its durability under cyclic mechanical stress. In contrast, the conventional serpentine structure achieves a significantly improved elongation at break of 28.42% (enhanced flexibility), yet its tensile strength decreases to 12.08 MPa, leading to insufficient mechanical stability under heavy vehicle loads. Notably, the novel serpentine structure strikes a balance in performance: while sacrificing a portion of flexibility (elongation at break = 17.46%), it boosts the tensile strength to 21.97 MPa, representing an 81.8% improvement over the conventional serpentine structure. Combined with the inherent stress-dispersing effect of the serpentine geometry, this enhanced strength effectively mitigates structural fatigue under repeated vehicle loading, ensuring long-term reliability for tunnel pavement applications. The shear mechanical properties of conventional and novel serpentine structures with different MXene contents were further evaluated (Figure 4b,c).

The piezoelectric output of the 0.75 wt% MXene/PVDF serpentine-structure sensor was characterized under periodic impact test conditions (frequency = 2 Hz, acceleration = 0.5 m/s^2^, sampling rate = 1000). Figure 4d,e display the *V*_oc_ and *I*_sc_ of the sensor under normal forces ranging from 2 N to 40 N, respectively: both *V*_oc_ and *I*_sc_ increase monotonically with the applied external force. The pressure sensitivity of the sensor is defined as the slope of the voltage-pressure curve (Figure 4f), and the sensor exhibits a high piezoelectric sensitivity of 13.65 mV/kPa within the linear pressure range of 68.49–205.48 kPa.

The feasibility of the serpentine-structure MXene/PVDF sensor for intelligent tunnel lighting was demonstrated in a real-world tunnel environment (Figure 4g and Video S1): the sensor was embedded under the tunnel pavement to detect the mechanical pressure of passing vehicles, triggering the automatic activation of tunnel lighting upon pressure detection; once the vehicle passed, the lighting system automatically deactivated to eliminate unnecessary energy consumption during idle periods. To quantify the energy-saving effect, a control group (conventional tunnel lighting with 24/7 continuous operation) and an experimental group (intelligent lighting controlled by the MXene/PVDF sensor) were established, and the active energy consumption of both groups over 30 days was monitored (Figure S8). As shown in Figure 4h, the active energy consumption of the control group is independent of the presence of vehicles; in contrast, the experimental group only consumes electrical energy when vehicles are detected. Statistics of the total active energy consumption over 30 days (Figure 4i) reveal that the control group consumes 36,630.5 kWh, while the experimental group consumes only 1816.02 kWh, corresponding to a remarkable energy saving rate of 95.04%.

## 4. Conclusions

In summary, a high-sensitivity MXene/PVDF flexible piezoelectric sensor is fabricated via EHD printing combined with a serpentine-structure design, and successfully applied in vehicle-responsive intelligent tunnel lighting systems. The optimized 0.75 wt% MXene/PVDF composite films exhibit a high piezoelectric sensitivity of 18.09 mV/kPa in the pressure range of 17.69–176.93 kPa, and achieve an open-circuit voltage of 5.21 V and a short-circuit current of 46.15 nA under a normal force of 15 N, which are 8.14 and 9.12 times higher than those of pure PVDF devices, respectively. This remarkable improvement originates from the synergistic effect of EHD printing-induced electric field polarization and interfacial hydrogen bonding between MXene and PVDF, which effectively promotes the directional alignment of PVDF dipoles and increases the content of piezoelectrically active β-phase. Furthermore, the device maintains stable piezoelectric output without obvious attenuation after 3,000 cycles, demonstrating excellent mechanical durability and electrical stability.

Meanwhile, the serpentine structure designed in this work endows the sensor with excellent mechanical flexibility and robustness: compared with piezoelectric thin films and conventional serpentine structures, the novel serpentine structure achieves a favorable balance between tensile strength (21.97 MPa) and elongation at break (17.46%), which can effectively disperse stress under repeated vehicle loading and ensure the long-term reliability of pavement integration. Field tests in actual tunnels further verify the practicability of the sensor-driven intelligent lighting system. By turning on tunnel lighting on demand only when vehicles are detected, the system achieves an energy-saving rate of 95.04% compared with the traditional lighting mode.

This work not only provides a feasible EHD printing strategy for the preparation of high-performance patterned piezoelectric composites, but also realizes the practical application of MXene/PVDF piezoelectric sensors in intelligent tunnel lighting. It offers a sustainable approach to the development of energy-efficient intelligent transportation infrastructure, and is of great significance for promoting the advancement of green transportation systems.

## Data Availability

The original contributions presented in this study are included in the article/Supplementary Materials. Further inquiries can be directed to the corresponding author.

## Supplementary Materials

The following supporting information can be downloaded at: https://www.mdpi.com/article/10.3390/ma19101976/s1, Figure S1: Schematic illustration of the intelligent sensing system for the tunnel lighting application based on MXene/PVDF flexible piezoelectric sensors; Figure S2: Electrohydrodynamic printing process of MXene/PVDF composites; Figure S3: Surface and cross-sectional SEM images of MXene/PVDF composite films with different MXene contents; Figure S4: X-ray photoelectron spectroscopy (XPS) survey of the as-etched MXene; Figure S5: X-ray diffraction (XRD) patterns of pure MXene, pure PVDF, and MXene/PVDF composite film; Figure S6: Piezoelectric output performance of MXene/PVDF composite films with different MXene contents under various normal forces; Figure S7: Piezoelectric output performance of MXene/PVDF composite films with different MXene contents under increasing normal force (0–40 N); Figure S8: Energy consumption monitoring of the intelligent sensing system for tunnel lighting based on MXene/PVDF flexible piezoelectric sensors; Figure S9: Comparison of piezoelectric performance between different materials; Figure S10: XPS characterization of pure PVDF and MXene/PVDF composites.; Figure S11: Long-term cyclic stability test of the optimized serpentine-structured MXene/PVDF flexible piezoelectric sensor over 10^5^ loading cycles; Table S1: Comparison of piezoelectric performance between different materials; Video S1: Real-time demonstration of vehicle-triggered lighting control for tunnel intelligent lighting. References [45,46,47,48,49] are cited in the Supplementary Materials.
