# Supplementary material for: A High-Sensitivity MXene/PVDF Flexible Piezoelectric Sensor for Intelligent Tunnel Lighting
Source: Materials (Basel). 2026 May 11;19(10):1976. doi: 10.3390/ma19101976 (PMC13208359; doi:10.3390/ma19101976)
Supplement: Supplementary file 1 [file materials-19-01976-s001.zip › materials-4230738-supplementary.zip!/materials-4230738-supplementary.pdf]

Supplemental information

A High-Sensitivity MXene/PVDF Flexible Piezoelectric Sensor for Intelligent Tunnel Lighting

Xi Xiong[1], Long Jin[1,*], Shenglong Wang[1], Tianpei Xu[1], Jiabin Zhang[1], Longchao Huang[1], Yong Ao[1], Weili Deng[1] and Weiqing Yang[1,2]

Supplementary Notes

Note S1

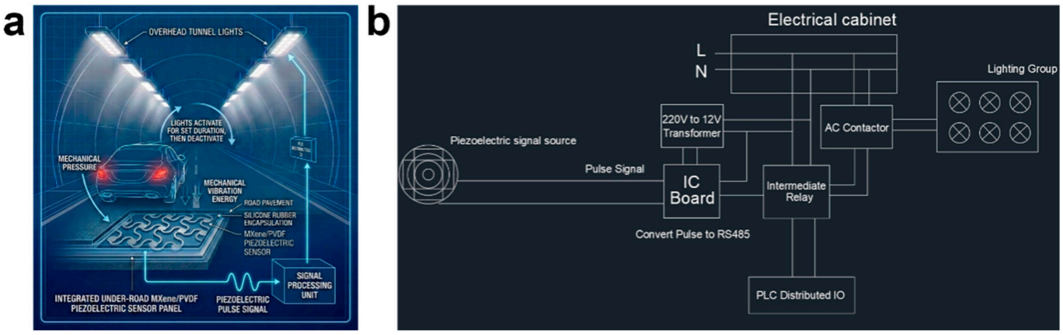

Figure S1 Schematic illustration of the intelligent sensing system for the tunnel lighting application based on MXene/PVDF flexible piezoelectric sensors. a Working principle of the piezoelectric sensing system in tunnel lighting application. **b** Electrical control block diagram of the intelligent lighting system.

The schematic illustration of the intelligent sensing system for tunnel lighting applications based on MXene/PVDF flexible piezoelectric sensors was presented (Figure S1). As shown in Figure S1a, when a vehicle travels over the road surface in the piezoelectric device integration area, mechanical pressure is converted into piezoelectric signals by the under-road MXene/PVDF piezoelectric sensor panel, which triggers the lighting system for vehicle detection and activation. Figure S1b depicts the electrical control block diagram of the intelligent lighting system, where the pulse signals generated by the piezoelectric signal source are processed by the IC board, transmitted to the PLC via RS485 communication, and subsequently control the AC contactor to realize the intelligent switching of the lighting group. These results demonstrate the feasibility of the proposed intelligent sensing system for tunnel lighting, which can achieve energy-saving and intelligent control by responding to vehicle-induced piezoelectric signals. In addition, the flexible MXene/PVDF piezoelectric sensors integrated into the road pavement provide opportunities for the practical application and large-scale deployment of such intelligent tunnel lighting systems.

Note S2

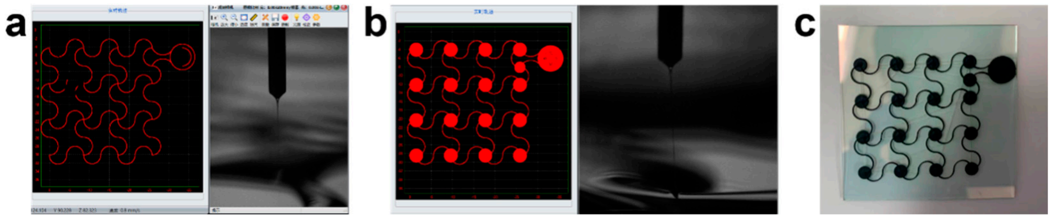

Figure S2 Electrohydrodynamic printing process of MXene/PVDF composites. a Photograph of EHD printing for the conventional serpentine structure, showing a stable Taylor cone. **b** Photograph of EHD printing for the novel serpentine structure, showing a stable Taylor cone. **c** Photograph of the as-printed MXene/PVDF composite on the ITO conductive glass substrate.

EHD printing process of MXene/PVDF composites is characterized (Figure S2). As shown in Figure S2a,b, stable Taylor cones are successfully formed during the EHD printing of the conventional and novel serpentine structures, respectively, which confirms the excellent processability and print-ability of the MXene/PVDF composite ink. The well-defined Taylor cone morphology is critical for achieving high-precision patterning of the serpentine electrodes. Figure S2c presents the photograph of the as-printed MXene/PVDF composite with the novel serpentine structure on the ITO conductive glass substrate, demonstrating the clear and continuous patterning capability of the EHD printing technique. These results verify the feasibility of fabricating patterned MXene/PVDF composites via EHD printing, which lays a solid foundation for the subsequent preparation of flexible piezoelectric sensors with tailored serpentine structures.

Note S3

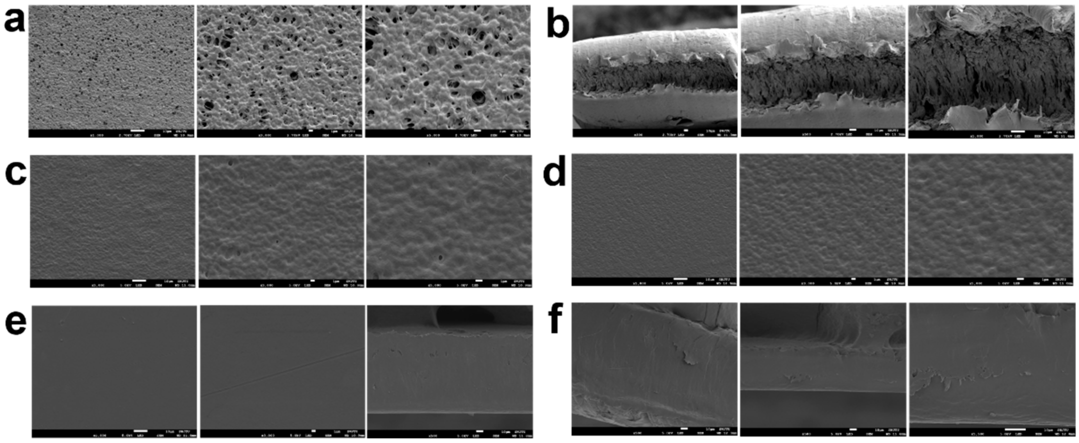

Figure S3 Surface and cross-sectional SEM images of MXene/PVDF composite films with different MXene contents. **a** Surface SEM images of pure PVDF film at different magnifications. **b** Cross-sectional SEM images of pure PVDF film at different magnifications. **c** Surface SEM images of MXene/PVDF composite film with 0.25 wt% MXene content at different magnifications. **d** Surface SEM images of MXene/PVDF composite film with 0.50 wt% MXene content at different magnifications. **e** Surface (left, middle) and cross-sectional (right) SEM images of MXene/PVDF composite film with 0.75 wt% MXene content at different magnifications. **f** Cross-sectional SEM images of MXene/PVDF composite films with 1.0 wt% (left) and 1.25 wt% (middle, right) MXene contents at different magnifications.

The surface and cross-sectional morphologies of pure PVDF and MXene/PVDF composite films with different MXene contents were systematically characterized by scanning electron microscopy (SEM), as presented in Figure S3. As shown in Figure S3a, the surface of the pure PVDF film exhibits a porous structure with uniformly distributed micropores, and the corresponding cross-sectional images (Figure S3b) reveal a distinct layered morphology with clear phase separation. For the MXene/PVDF composite films, the surface morphologies evolve with the increase of MXene content. The composite film with 0.25 wt% MXene (Figure S3c) shows a relatively smooth surface with reduced porosity compared to pure PVDF, while the 0.50 wt% MXene film (Figure S3d) displays a slightly rougher surface with more pronounced microstructures. The 0.75 wt% MXene film (Figure S3e) exhibits a dense and uniform surface (left and middle panels) and a compact cross-sectional structure (right panel) without obvious agglomeration. For higher MXene contents (1.0 wt% and 1.25 wt%, Figure S3f), the cross-sectional images show a more pronounced layered structure with increased filler dispersion, indicating the successful incorporation of MXene nanosheets into the PVDF matrix. These morphological changes suggest that the MXene content significantly influences the microstructure of the composite films, which is crucial for optimizing their piezoelectric and mechanical properties.

Note S4

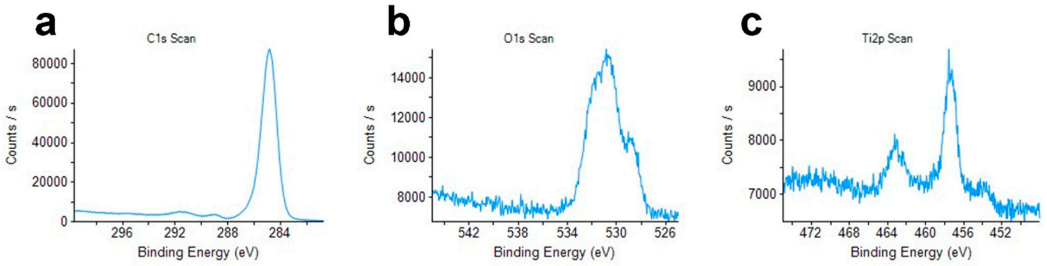

Figure S4 X-ray photoelectron spectroscopy (XPS) survey of the as-etched MXene. a XPS spectrum of C 1s for the MXene film. **b** XPS spectrum of O 1s for the MXene film. **c** XPS spectrum of Ti 2p for the MXene film.

The chemical composition and surface elemental states of the as-etched MXene, which was obtained from the MAX phase and drop-cast from an MXene/DMF solution, were characterized by X-ray photoelectron spectroscopy (XPS). As shown in Figure S4, the high-resolution XPS spectra of C 1s, O 1s, and Ti 2p were collected to confirm the successful etching of the MAX phase and the formation of MXene. The C 1s spectrum (Figure S4a) exhibits a dominant peak at ~284.8 eV, corresponding to the C-C bonds in the MXene structure. The O 1s spectrum (Figure S4b) shows a strong peak at ~532.0 eV, which can be attributed to the surface oxygen-containing functional groups introduced during the etching process. The Ti 2p spectrum (Figure S4c) displays two characteristic peaks at ~455.5 eV and ~461.5 eV, consistent with the Ti-C and Ti-O bonds in MXene, confirming the successful preparation of the MXene nanosheets.

Note S5

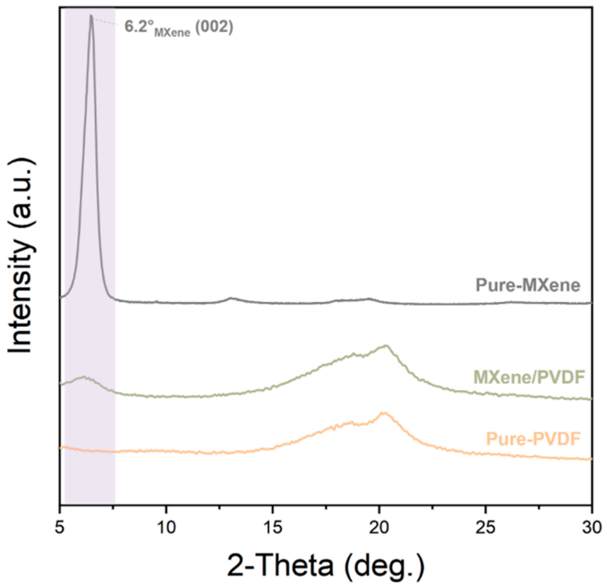

Figure S5 X-ray diffraction (XRD) patterns of pure MXene, pure PVDF, and MXene/PVDF composite film.

The crystalline structures of pure MXene, pure PVDF, and MXene/PVDF composite film were characterized by X-ray diffraction (XRD), as presented in Figure S5. The XRD pattern of pure MXene displays a distinct (002) diffraction peak at 2θ = 6.2°, which is a characteristic signature of the successfully exfoliated MXene nanosheets, indicating the removal of the Al layer from the MAX phase. For pure PVDF, a broad diffraction peak centered at 2θ = 20.3° is observed, corresponding to the α and β crystalline phases of PVDF. In the MXene/PVDF composite film, the characteristic peak of MXene at 6.2° is not prominent, which can be attributed to the low loading content of MXene and the good dispersion of MXene nanosheets within the PVDF matrix. Meanwhile, the diffraction peak of PVDF in the composite film shows a slight shift and intensity change compared to pure PVDF, suggesting the interaction between MXene and PVDF, which may influence the crystalline structure of PVDF. These results confirm the successful preparation of the MXene/PVDF composite film and the effective integration of MXene nanosheets into the PVDF matrix.

Note S6

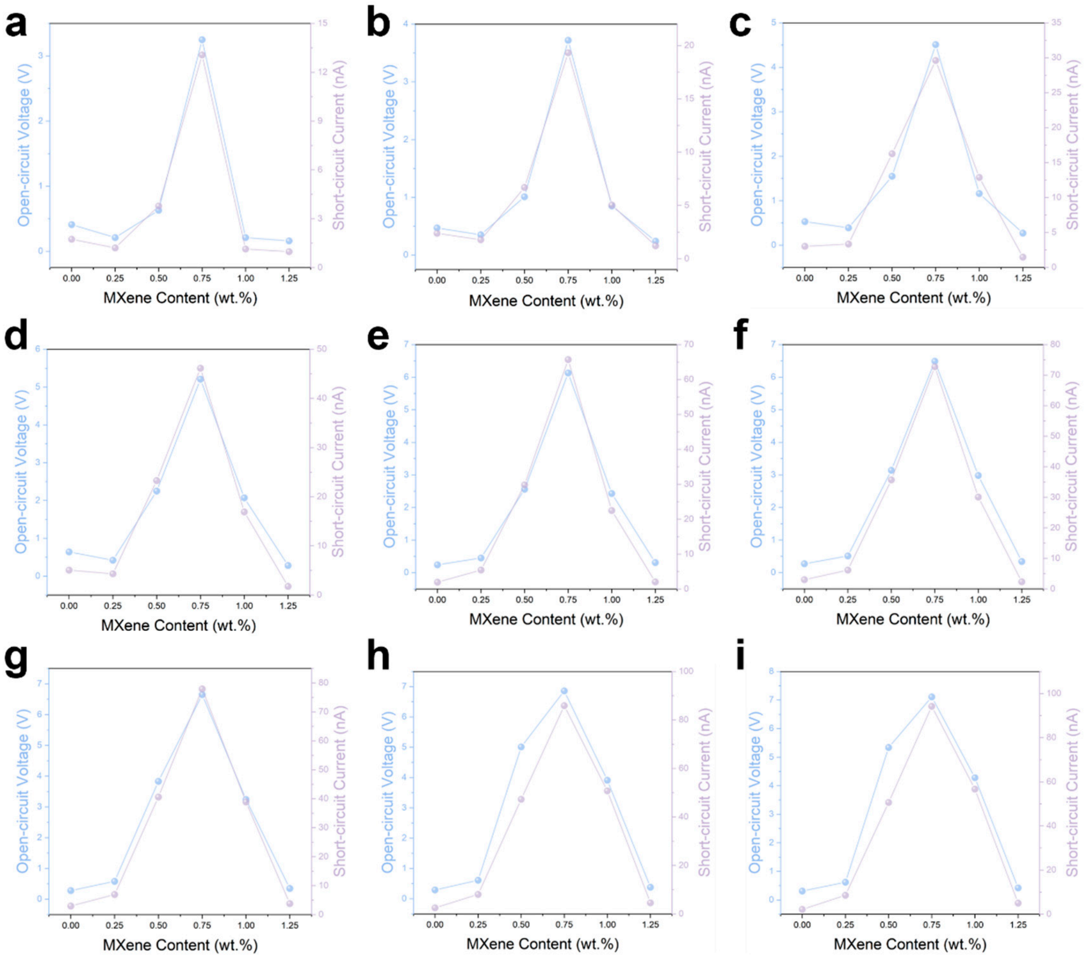

Figure S6 Piezoelectric output performance of MXene/PVDF composite films with different MXene contents under various normal forces. **a** Piezoelectric output of MXene/PVDF composite film under a force of 2 N. **b** Piezoelectric output of MXene/PVDF composite film under a force of 5 N. **c** Piezoelectric output of MXene/PVDF composite film under a force of 10 N. **d** Piezoelectric output of MXene/PVDF composite film under a force of 15 N. **e** Piezoelectric output of MXene/PVDF composite film under a force of 20 N. **f** Piezoelectric output of MXene/PVDF composite film under a force of 25 N. **g** Piezoelectric output of MXene/PVDF composite film under a force of 30 N. **h** Piezoelectric output of MXene/PVDF composite film under a force of 35 N. **i** Piezoelectric output of MXene/PVDF composite film under a force of 40 N.

The piezoelectric output performance of MXene/PVDF composite films with different MXene contents (0.00, 0.25, 0.50, 0.75, 1.00, and 1.25 wt%) was systematically evaluated under various normal forces ranging from 2 N to 40 N (Figure S6). As shown in all subplots (Figure S6a–i), both the V_{oc} and I_{sc} of the composite films exhibit a volcano-shaped trend with the increase of MXene content, first increasing and then decreasing. Notably, the composite film with 0.75 wt% MXene consistently delivers the highest V_{oc} and I_{sc} across all tested force levels, indicating that this MXene loading is the optimal doping ratio for maximizing the piezoelectric response. This enhancement can be attributed to the improved stress

transfer and charge carrier transport at this specific content, while excessive MXene loading (≥1.00 wt%) leads to agglomeration and reduced piezoelectric performance. Additionally, the piezoelectric output of all composite films increases monotonically with the applied force, which is consistent with the fundamental piezoelectric effect, where higher mechanical stress induces greater charge separation and thus a stronger electrical output. These results confirm the robust piezoelectric performance of the 0.75 wt% MXene/PVDF composite film under a wide range of mechanical forces, making it a promising candidate for flexible piezoelectric sensor applications.

Note S7

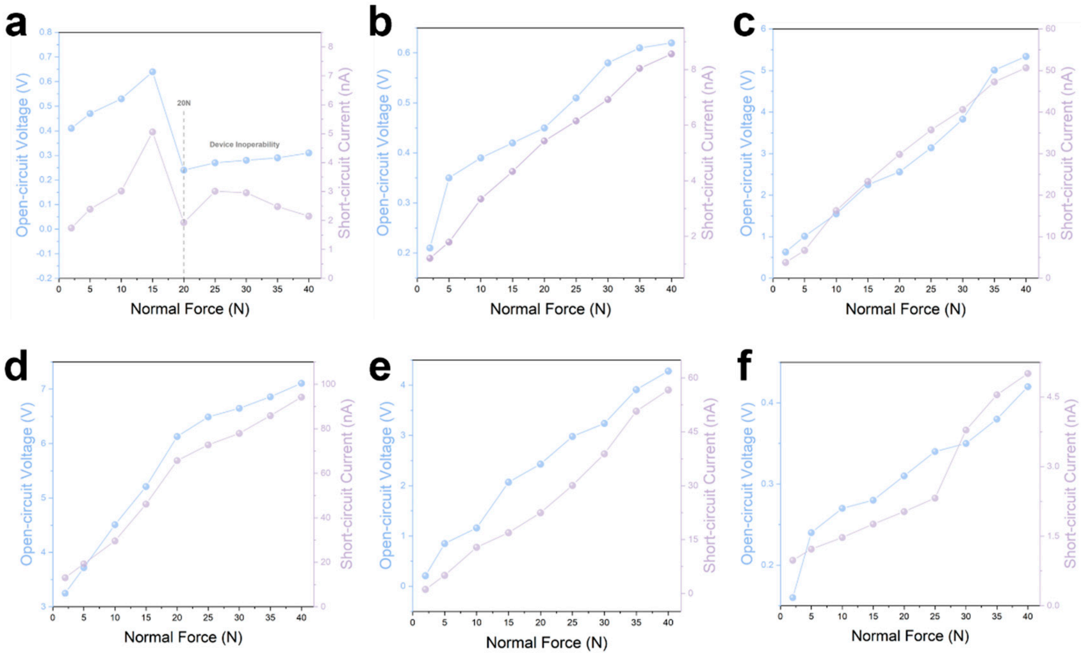

Figure S7 Piezoelectric output performance of MXene/PVDF composite films with different MXene contents under increasing normal force (0–40 N). a Piezoelectric output of pure PVDF composite film. **b** Piezoelectric output of MXene/PVDF composite film with 0.25 wt% MXene. **c** Piezoelectric output of MXene/PVDF composite film with 0.50 wt% MXene. **d** Piezoelectric output of MXene/PVDF composite film with 0.75 wt% MXene. **e** Piezoelectric output of MXene/PVDF composite film with 1.00 wt% MXene. **f** Piezoelectric output of MXene/PVDF composite film with 1.25 wt% MXene.

The piezoelectric output performance of pure PVDF and MXene/PVDF composite films with different MXene contents (0.25, 0.50, 0.75, 1.00, and 1.25 wt%) was further investigated under varying normal forces ranging from 0 to 40 N (Figure S7). As shown in Figure S7a, the pure PVDF film exhibits a gradual increase in V_{oc} and I_{sc} with increasing force up to 15 N, followed by a sudden drop in both signals at 20 N (176.93 kPa), indicating device inoperability. This failure can be attributed to the inherent micro-structural defects (e.g., porous and layered morphology) observed in the SEM images of pure PVDF (Figure S3), which lead to mechanical instability and structural breakdown under moderate mechanical stress. In contrast, all MXene/PVDF composite films (Figure S7b–f) maintain stable piezoelectric output with monotonically increasing V_{oc} and I_{sc} across the entire force range (0–40 N), demonstrating significantly improved mechanical robustness and structural integrity compared to pure PVDF. Among all composite films, the 0.75 wt% MXene/PVDF sample (Figure S7d) delivers the highest piezoelectric output, consistent with the previous results (Figure S6), confirming its optimal performance. The enhanced mechanical stability of the composite films can be ascribed to the reinforcing effect of MXene nanosheets, which mitigate the micro-structural defects and improve the stress distribution within the PVDF matrix. These results highlight the critical role of

MXene incorporation in enhancing both the piezoelectric performance and mechanical durability of the composite films, making them suitable for practical sensor applications under varying mechanical loads.

Note S8

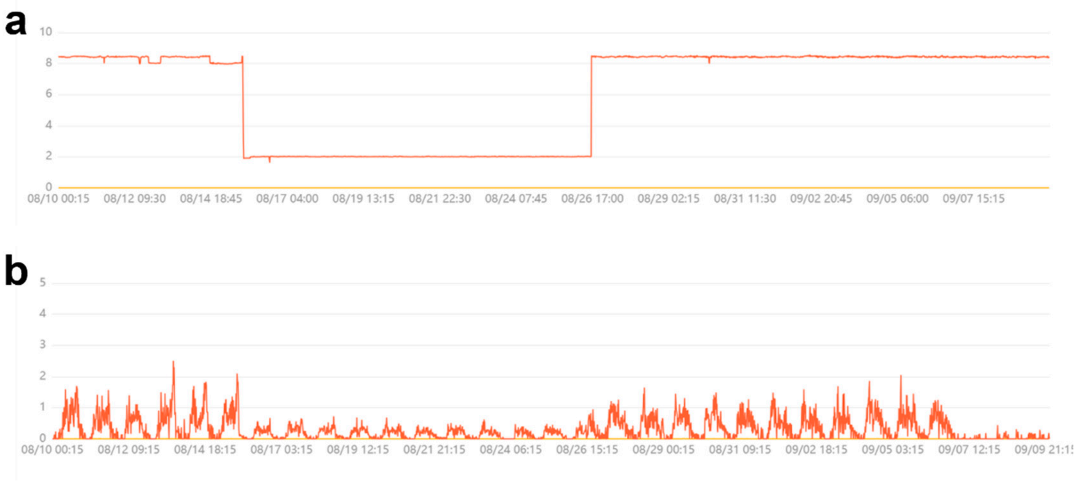

Figure S8 Energy consumption monitoring of the intelligent sensing system for tunnel lighting based on MXene/PVDF flexible piezoelectric sensors. **a** Daily energy consumption of the control group, monitored by smart electricity meters. **b** Daily energy consumption of the experimental group, monitored by smart electricity meters.

The energy-saving performance of the vehicle-responsive tunnel lighting system, enabled by the novel serpentine-structure MXene/PVDF flexible piezoelectric sensor, was validated in a real-world tunnel environment (Figure S8). The system operates on an on-demand principle: the under-road piezoelectric sensor detects the mechanical pressure of passing vehicles, triggering the lighting system to activate. Once the vehicle has passed, the lights automatically deactivate, eliminating unnecessary energy consumption during idle periods. A control group (conventional tunnel lighting with continuous, 24/7 operation) and an experimental group (intelligent lighting controlled by the MXene/PVDF sensor) were established for direct comparison. The daily energy consumption of both groups was continuously recorded by smart electricity meters. The monitoring data clearly shows that the experimental group achieves a drastically reduced energy consumption profile compared to the control group. This significant reduction is attributed to the elimination of constant lighting when no vehicles are present, directly translating the intelligent vehicle detection into tangible energy savings. These results confirm the practical efficacy of the MXene/PVDF-based on-demand lighting system, which not only delivers substantial energy savings but also represents a critical step forward in the development of smart transportation infrastructure. By seamlessly integrating flexible piezoelectric sensors into roadways, this technology paves the way for more energy-efficient, responsive, and sustainable green transportation systems, contributing to global efforts in carbon reduction and the construction of eco-friendly smart cities.

Note S9

(i) A comparison graph showing performance trends across different materials

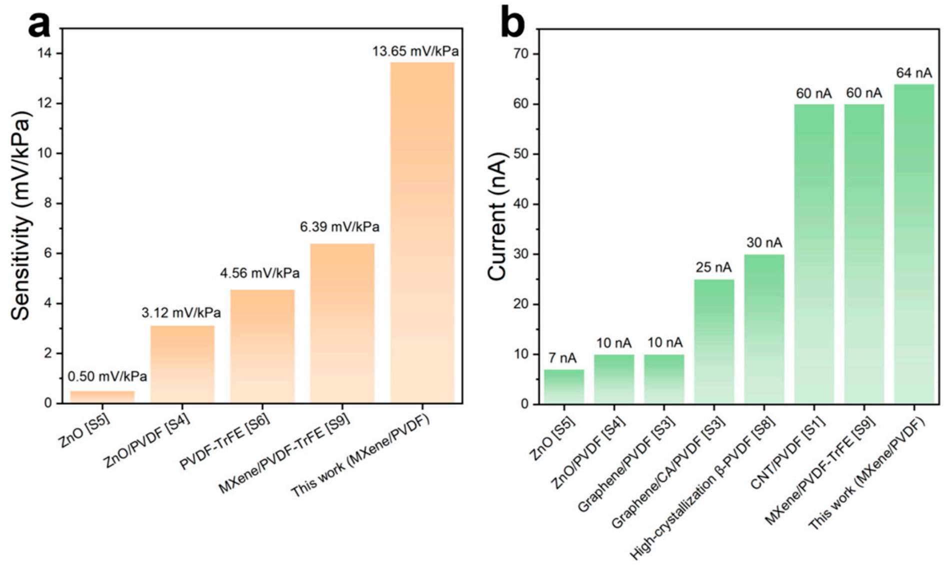

Figure S9 Comparison of piezoelectric performance between different materials. a piezoelectric sensitivity of different materials. **b** output current of different materials.

The piezoelectric performance of the MXene/PVDF sensor was compared with other representative flexible piezoelectric materials (Figure S9 and Table S1). Our device exhibits the highest sensitivity (13.65 mV/kPa) and output current (64 nA) under the same test conditions, which is significantly improved compared to traditional materials such as ZnO and pure PVDF derivatives, and also outperforms most reported MXene-based piezoelectric composites. This excellent performance is attributed to the synergistic effect of MXene and PVDF, as well as the optimized serpentine structure, demonstrating its great potential for practical applications such as intelligent tunnel lighting.

(ii) A summary table compiling piezoelectric performance metrics of related systems

Table S1 Comparison of piezoelectric performance between different materials

Nanocomposite	Sensitivity	Output current (nA)	References
CNT/PVDF	0.056 V N^{-1}	60	[S1]
MXene/PVDF	0.048 V N^{-1}	/	[S2]
Graphene/PVDF	/	10	[S3]
Graphene/CA/PVDF	/	25	[S3]

ZnO/PVDF	3.12 mV kPa^{-1}	10	[S4]
ZnO	0.50 mV kPa^{-1}	7	[S5]
PVDF-TrFE	4.56 mV kPa^{-1}	/	[S6]
$GaFeO_3$/PVDF	/	4	[S7]
High-crystallization β-PVDF	/	30	[S8]
MXene/PVDF-TrFE	6.39 mV kPa^{-1}	60	[S9]
This work	13.65 mV kPa^{-1}	64	/

Supplementary References

[S1] Zeng W, Deng W, Yang T, et al. Gradient CNT/PVDF piezoelectric composite with enhanced force-electric coupling for soccer training[J]. *Nano Research*, **2023**, 16(8): 11312-11319. https://doi.org/10.1007/s12274-023-5869-6

[S2] Zhao Q, Yang L, Ma Y, et al. Highly sensitive, reliable and flexible pressure sensor based on piezoelectric PVDF hybrid film using MXene nanosheet reinforcement[J]. *Journal of Alloys and Compounds*, **2021**, 886: 161069. https://doi.org/10.1016/j.jallcom.2021.161069

[S3] Shu K, Li W, Zhang Y, et al. An rGO/cellulose acetate/PVDF piezoelectric separator for mitigating self-discharge in supercapacitor[J]. *Chemical Engineering Journal*, **2025**: 166056. https://doi.org/10.1016/j.cej.2025.166056

[S4] Wang Z, Sun Y, Wang S, et al. Insight into piezoelectricity modulation mechanism of ZnO doped with Y ions[J]. *Journal of Materials Chemistry A*, **2024**, 12(21): 12435-12442. https://doi.org/10.1039/D4TA01317D

[S5] Tian G, Xiong D, Su Y, et al. Understanding the potential screening effect through the discretely structured ZnO nanorods piezo array[J]. *Nano Letters*, **2020**, 20(6): 4270-4277. https://doi.org/10.1021/acs.nanolett.0c00793

[S6] Tian G, Tang L, Zhang J, et al. Ultrathin epidermal P (VDF-TrFE) piezoelectric film for wearable electronics[J]. *ACS Applied Electronic Materials*, **2023**, 5(3): 1730-1737. https://doi.org/10.1021/acsaelm.2c01760

[S7] Mishra M, Roy A, Dash S, et al. Flexible nano-GFO/PVDF piezoelectric-polymer nano-composite films for mechanical energy harvesting[C]//IOP Conference Series: *Materials Science and Engineering*. IOP Publishing, **2018**, 338(1): 012026. https://doi.org/10.1088/1757-899X/338/1/012026

[S8]Jin L, Ma S, Deng W, et al. Polarization-free high-crystallization β-PVDF piezoelectric nanogenerator toward self-powered 3D acceleration sensor[J]. *Nano Energy*, **2018**, 50: 632-638. https://doi.org/10.1016/j.nanoen.2018.05.068

[S9] Ao Y, Jin L, Wang S, et al. Dual structure reinforces interfacial polarized MXene/PVDF-TrFE piezoelectric nanocomposite for pressure monitoring[J]. *Nano-Micro Letters*, **2025**, 17(1): 320. https://doi.org/10.1007/s40820-025-01839-5

Note S10

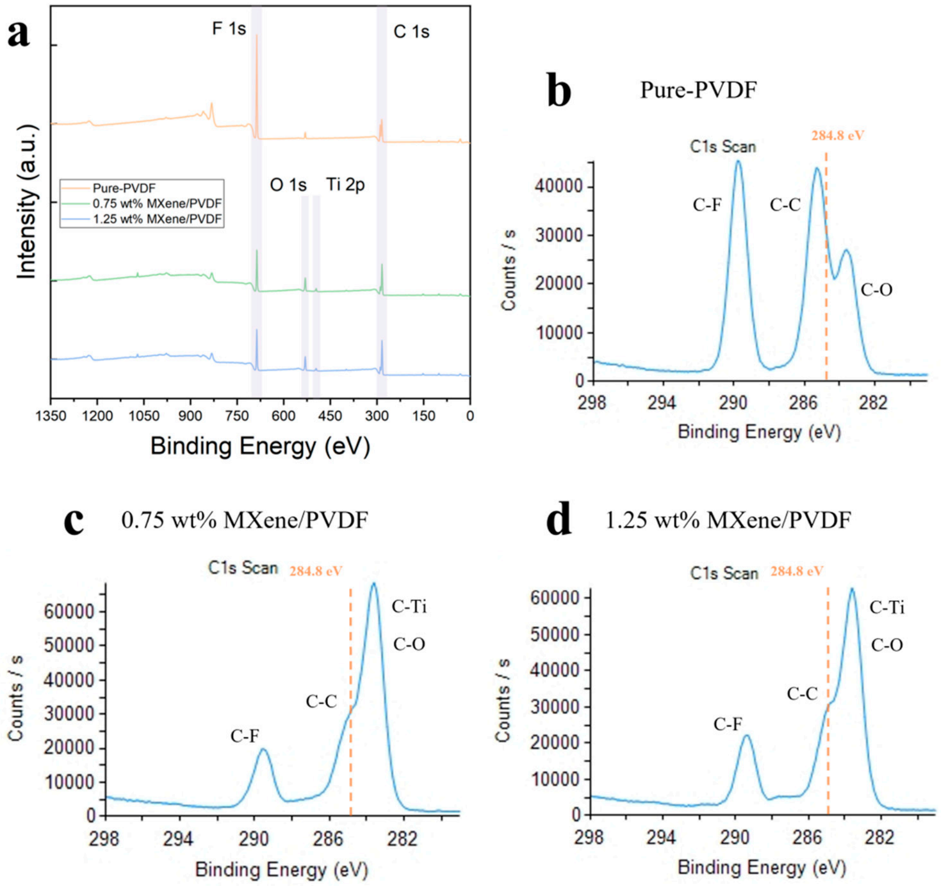

Figure S10 XPS characterization of pure PVDF and MXene/PVDF composites. a Full XPS survey spectra of pure PVDF, 0.75 wt% MXene/PVDF, and 1.25 wt% MXene/PVDF composites. **b-d** High-resolution C 1s XPS spectra of (b) pure PVDF, (c) 0.75 wt% MXene/PVDF, and (d) 1.25 wt% MXene/PVDF composites.

The full XPS survey spectra in Figure S10 a confirm the successful introduction of MXene into the PVDF matrix. Compared with pure PVDF, the MXene/PVDF composites exhibit clear Ti 2p signals at ~460 eV, verifying the presence of $Ti_3C_2T_x$ MXene in the films, while the consistent F 1s signal across all samples confirms the integrity of the PVDF matrix.

The high-resolution C 1s spectra in Figure S10 b-d provide direct evidence of strong interfacial interactions between MXene and PVDF. For pure PVDF, the C-C peak is significantly shifted to a higher binding energy (left-shifted) relative to the standard C-C binding energy of 284.8 eV, which is attributed to the strong

electron-withdrawing effect of fluorine atoms in PVDF pulling electron density away from the C-C bonds, reducing their electron cloud density and increasing the binding energy. The weak C-O signal observed in pure PVDF is likely due to minor surface contamination.

For the MXene/PVDF composites, the C-C peak shifts back toward lower binding energy, closer to 284.8 eV. This shift is caused by the formation of hydrogen bonds between the -OH groups on MXene nanosheets and the fluorine atoms in PVDF, which weakens the electron-withdrawing effect of fluorine, restores the electron cloud density of C-C bonds, and thus reduces their binding energy. The presence of MXene is also reflected in the C 1s spectra, where signals corresponding to MXene-related C-Ti species appear in the composites, confirming the coexistence of MXene and PVDF at the measured surface.

These XPS results, especially the binding energy shift of C-C bonds induced by hydrogen bonding between MXene and PVDF, provide direct spectroscopic evidence for the interfacial interactions that play a key role in promoting β-phase crystallization and enhancing piezoelectric performance.

Note S11

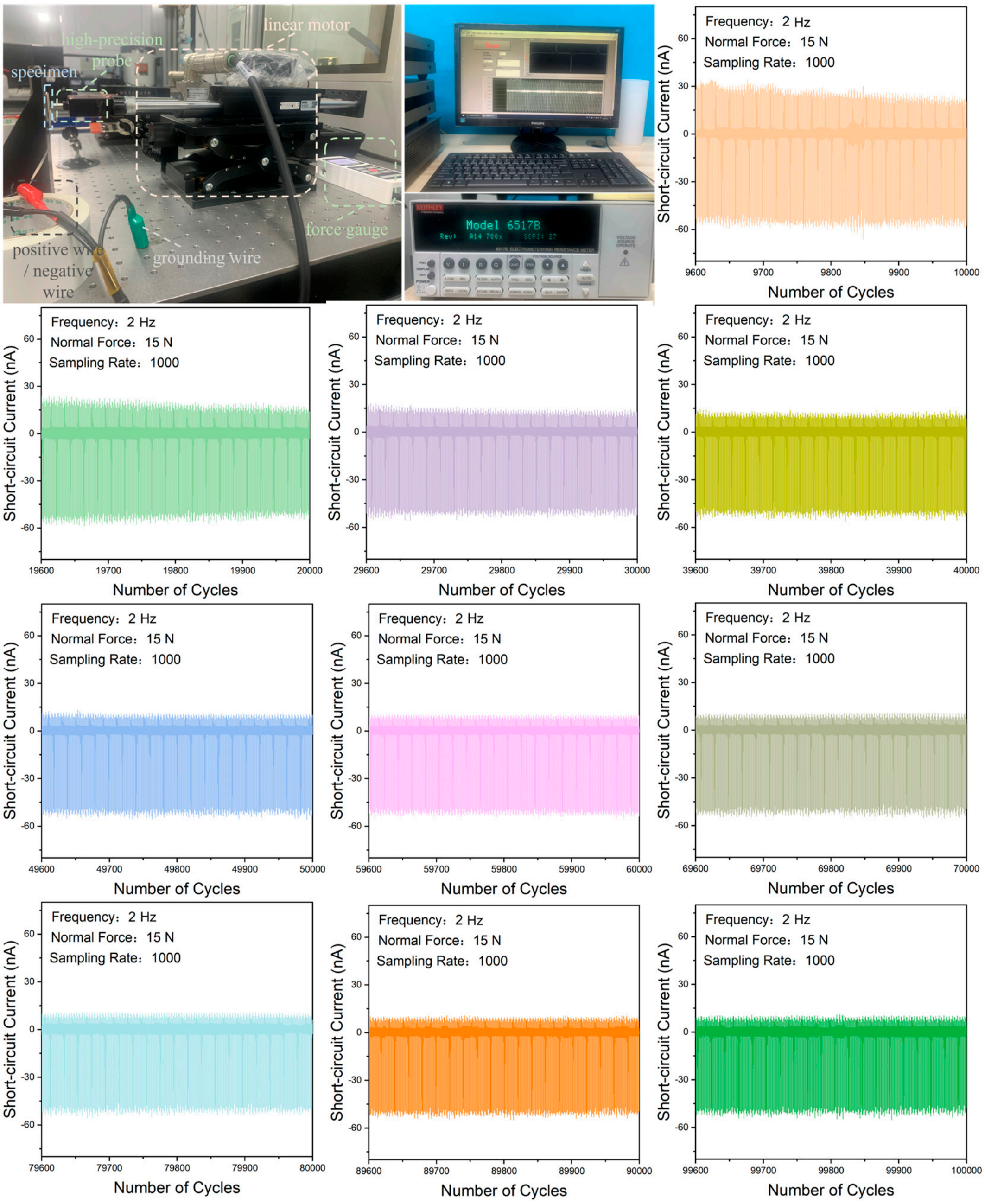

Figure S11 Long-term cyclic stability test of the optimized serpentine-structured MXene/PVDF flexible piezoelectric sensor over 10^5 loading cycles. The test was performed at a frequency of 2 Hz with an applied normal force of 15 N, showing the short-circuit current output recorded at different cycle intervals (from 10^4 to 10^5 cycles).

The test was conducted under conditions relevant to practical infrastructure scenarios: an applied normal force of 15 N at a frequency of 2 Hz, for a total of 10^5 continuous cycles. As shown in **Figure S11**, the short-circuit current output of the

sensor remains highly stable throughout the entire test duration, with no obvious attenuation or fluctuation in signal amplitude across different cycle intervals. This confirms that our serpentine-structured MXene/PVDF composite can withstand at least 10^5 loading cycles without performance degradation, satisfying the basic durability requirements for infrastructure applications and demonstrating outstanding fatigue resistance.

This excellent long-term stability is attributed to the combined advantages of our material and structural design: the serpentine geometry uniformly distributes cyclic stress across the film, eliminating stress concentration that would otherwise lead to microcrack initiation and propagation; meanwhile, the strong interactions between MXene and PVDF help to keep the composite structure intact under repeated loading, ensuring stable piezoelectric response throughout the test.
